# The Inhibitory Activity of Curcumin on P-Glycoprotein and Its Uptake by and Efflux from LS180 Cells Is Not Affected by Its Galenic Formulation

**DOI:** 10.3390/antiox10111826

**Published:** 2021-11-17

**Authors:** Sandra Flory, Romina Männle, Jan Frank

**Affiliations:** Department of Food Biofunctionality, Institute of Nutritional Sciences, University of Hohenheim, 70599 Stuttgart, Germany; sandra.flory@nutres.de (S.F.); romina.maennle@gmx.de (R.M.)

**Keywords:** cellular uptake, curcuminoids, cyclodextrin complex, drug interactions, efflux transporter, intestinal cell line, multidrug resistance protein 1, polysorbate 80 micelles, turmeric oils

## Abstract

The biological activities of curcumin in humans, including its antioxidative and anti-inflammatory functions, are limited by its naturally low bioavailability. Different formulation strategies have been developed, but the uptake of curcumin from these galenic formulations into and efflux from intestinal cells, which may be critical processes limiting bioavailability, have not been directly compared. Furthermore, little is known about their effect on P-glycoprotein activity, an important determinant of the pharmacokinetics of potentially co-administered drugs. P-glycoprotein activity was determined in LS180 cells, incubated with 30 or 60 µmol/L of curcumin in the form of seven different formulations or native curcuma extract for 1 h. All formulations inhibited P-glycoprotein activity at both concentrations. Curcumin uptake, after 1 h incubation of LS180 cells with the formulations (60 µmol/L), showed significant variability but no consistent effects. After 1 h pre-treatment with the formulations and further 8 h with curcumin-free medium, curcumin in cell culture supernatants, reflecting the efflux, differed between individual formulations, again without a clear effect. In conclusion, curcumin inhibits P-glycoprotein activity independently of its formulation. Its uptake by and efflux from intestinal cells was not significantly different between formulations, indicating that these processes are not important regulatory points for its bioavailability.

## 1. Introduction

The lipophilic phytochemical curcumin is the main curcuminoid from *Curcuma longa Linn.* and has been used in Ayurvedic medicine due to its many reported health beneficial activities, including anticarcinogenic, anti-inflammatory, and anti-diabetic effects [1]. The direct antioxidative activities of curcumin have been reported to be 2.5-fold stronger than those of vitamin C and are facilitated by the free radical scavenging functions of its phenolic hydroxyl groups. Curcumin also acts as an indirect antioxidant by increasing the synthesis of the antioxidant peptide glutathione, as well as the expression and activities of antioxidative enzymes, such as superoxide dismutase, catalase and glutathione peroxidase (reviewed in [2,3,4]).

More recently, curcumin has been investigated as a nutraceutical in the prevention and complementary therapy of diseases, such as cancer, chronic inflammatory disorders, Alzheimer’s disease, and many more (reviewed in [1,5,6,7]). The central limitation of its application is its naturally low bioavailability that results in no or small detectable plasma concentrations after oral administration [8]. Curcumin in its native form is lipophilic and poorly soluble in water. The little amount that is absorbed is rapidly metabolized in intestinal and hepatic cells. Curcumin is reduced to dihydro-, tetrahydro-, hexahydro-, or octahydrocurcumin, and the parent compound and the reduced metabolites are conjugated with glucuronic acid or sulfate by UDP-glucuronosyltransferases and sulfotransferases, respectively. The conjugates are shuttled out of the cell by efflux transporters, such as P-glycoprotein [9,10]. Several mechanistic approaches to combat the problem of low bioavailability have been investigated. In previous pharmacokinetic human trials, enhancing the water solubility and increasing digestive stability, especially by incorporation of curcumin into polysorbate 80 (PS80) micelles, or γ-cyclodextrin complexes, have been successful bioavailability-enhancing strategies [11,12,13]. Other, less effective approaches are the formulation of curcumin into phospholipid bilayers, in the form of phytosomes or liposomes [14,15], into nanoparticles with gum ghatti and glycerin [16,17] or the addition of adjuvants, such as piperine or turmeric oils, that aim to reduce curcumin metabolism and elimination [18,19,20].

The efflux transporter P-glycoprotein (P-gp) is a 170-kDa transmembrane protein, encoded by the *mdr1* gene, member of the family of ATP-binding cassette transporters, and is expressed in the liver, intestine, and kidney. Its overexpression in cancer cells is the main cause of multidrug resistance [21]. Intestinal P-gp transports xenobiotics, such as drugs or phytochemicals, including curcumin, back into the intestinal lumen and consequently influences their bioavailability [9,22]. Previously, curcumin, in its native form, has been shown to inhibit the activity and the expression of P-gp in intestinal [23,24,25,26,27] and multidrug-resistant cells [28,29,30,31,32,33], thereby influencing the pharmacokinetics of co-administered drugs [24,30,34]. Curcumin and its glucuronides do not only affect P-gp but, vice versa, the activity of P-gp and other efflux transporters affect curcumin accumulation and efflux from cultured intestinal epithelial cells (LS180 and Caco-2). For example, the addition of specific inhibitors, such as turmeric oils or phytochemicals, inhibited P-gp or breast cancer resistance protein and, in parallel, decreased efflux of curcumin or its glucuronide from Caco-2 cells [9,18]. The impact of different formulation strategies, aiming at the improvement of curcumin bioavailability on its intestinal uptake into and efflux from intestinal epithelial cells and on the activity of efflux transporters, have not been investigated yet.

In a preceding human trial, we investigated the pharmacokinetics of curcumin, from seven different galenic formulations relative to that of native curcumin, and observed that only those with PS80 and γ-cyclodextrin significantly improved its bioavailability [13]. Here, we studied the effects of these curcumin formulations and native curcumin on P-gp activity, as well as curcumin uptake, into and efflux from LS180 cells.

## 2. Materials and Methods

### 2.1. Materials

The following curcumin formulations were used for experiments: A native curcuma extract (150 mmol/L in DMSO; Jupiter Ley, Okkal, India), liposomal curcumin (75 mmol/L in H_2_Odd; Longvida^®^; Verdure Sciences, Noblesville, IN, USA), a curcuma extract with turmeric oils (150 mmol/L in DMSO; BCM-95^®^; Arjuna Natural Extracts, Kerala, India), a curcuma extract with adjuvants (75 mmol/L in DMSO; TISSO Naturprodukte GmbH, Wenden, Germany), submicron-particle curcumin (150 mmol/L in H_2_Odd; Theracurmin CR-043P; Theravalues Corp., Tokyo, Japan), phytosomal curcumin (50 mmol/L in H_2_Odd; Meriva^®^; Indena S.p.A., Milan, Italy) a curcumin-γ-cyclodextrin complex (75 mmol/L in H_2_Odd; Cavacurmin^®^; Wacker Chemie, Munich, Germany) and curcumin incorporated into PS80 micelles consisting of 93% PS80 and 7% curcuminoid powder (30 mmol/L in H_2_Odd; NovaSOL^®^ Curcumin; AQUANOVA AG, Darmstadt, Germany). For experiments, rhodamine 123 (R123; stock 1 mg/mL in ethanol), verapamil hydrochloride (stock 50 mmol/L in H_2_Odd), elacridar (stock 2 mg/mL in DMSO) and PS80 were purchased from Sigma-Aldrich (Steinheim, Germany). Empty PS80 micelles were from AQUANOVA AG (Darmstadt, Germany). β-Glucuronidase type H-1 from *Helix pomatia* (EC 3.2.1.31) was purchased from Sigma-Aldrich (Taufkirchen, Germany). Methanol, ethylacetate, ethanol and acetonitrile were HPLC grade.

### 2.2. Cell Culture Conditions

All experiments were conducted in LS180 cells, a human colorectal adenocarcinoma cell line (American Type Culture Collection, Manassas, VA, USA), between passages 46 and 51. This cell line is recommended for studies on the regulation of P-gp and other efflux proteins [35]. Cells were cultured in Eagle’s minimum essential medium (MEM, Sigma-Aldrich, Steinheim, Germany), supplemented with 10% fetal calf serum (Life Technologies Corporation, Gran Island, NE, USA), 1% non-essential amino acids (Carl Roth GmbH, Karlsruhe, Germany), 1% sodium pyruvate (Carl Roth, Karlsruhe, Germany) and 1% penicillin/streptomycin (Biochrom GmbH, Berlin, Germany) at 5% CO_2_ and 37 °C.

### 2.3. Cell Viability

To ensure detectable amounts of curcumin in cell lysates and supernatants, in all experiments, formulations were normalized to the maximum curcumin concentration that did not show cytotoxic effects. To determine the appropriate concentration, neutral red uptake assays were conducted as described in Repetto et al., (2008) with minor modifications [36]. Then, 3 × 10^5^ cells/well were seeded in 48-well plates and grown for 24 h at 37 °C and 5% CO_2_. Cells were incubated with all formulations normalized to concentrations up to 75 µmol/L, a positive control (0.1% Triton X-100), solvent controls (DMSO, empty micelles, PS80), and a negative control (MEM Complete) for 1 h at 37 °C and 5% CO_2_. Then, substances were removed and cells were incubated with neutral red dye (Sigma-Aldrich, Taufkirchen, Germany) diluted to 140 µmol/L in MEM Complete for further 2 h at 37 °C and 5% CO_2_. The dye was discarded, and neutral red destain solution (50% ethanol, 49% H_2_Odd, 1% glacial acetic acid) was added and incubated for 15 min at room temperature while shaking. Absorbance was quantified with a Synergy HT (BioTek^®^ Instruments GmbH, Bad Friedrichshall, Germany) at 580 nm. Medium control served as a reference for 100% viability.

### 2.4. P-Glycoprotein Activity

The P-gp activity assay was conducted as described before with modifications [37]. Briefly, 3 × 10^5^ cells/well were seeded in 48-well plates and grown for 24 h. Half of the cells were incubated with the formulations normalized to 30 µmol/L or 60 µmol/L curcumin, solvent controls (0.12% or 0.06% DMSO, 0.04% or 0.02% PS80, and 0.04% or 0.02% empty PS80 micelles), negative control (MEM Complete), or positive control (100 µmol/L verapamil as in [38]) and 1 µg/mL R123 and 3.5 µmol/L elacridar, the other half with the test substances and 1 µg/mL R123 without elacridar for 1 h at 37 °C and 5% CO_2_. Then, cells were washed three times with ice-cold Hank’s balanced salt solution and lysed by an incubation with lysis buffer (0.8% NaOH, 0.5% Triton X-100, 4% protease inhibitor (cOmplete^TM^ Protease Inhibitor Coktail, Roche, Mannheim, Germany) for 10 min at room temperature while shaking, followed by a 30 min incubation at 37 °C. Fluorescence intensity was read by a Synergy HT) at emission/excitation 485/529 and was normalized to protein content (Pierce^TM^ BCA Protein-Assay, Thermo Fisher Scientific Inc., Rockford, IL, USA). Relative intracellular R123 was calculated by referring the quotient of fluorescence intensity with elacridar by fluorescence intensity without elacridar to the one from medium control cells.

### 2.5. Cellular Uptake Experiments

Additionally, 2 × 10^6^ cells/well were seeded in 6-well plates and grown for 24 h. Cells were treated with the formulations, normalized to 60 µmol/L curcumin, and medium control for 1 h at 37 °C and 5% CO_2_. For stability control, all solutions were incubated in parallel without cells. Aliquots were frozen at −80 °C before and after 1 h incubation. For the cellular uptake experiments, supernatants were frozen at −80 °C after 1 h incubation. Cells were washed with PBS, scratched off the plate, washed again with PBS and lysed by an incubation on ice with 20 µL lysis buffer ((150 mmol/L NaCl; 50 mmol/L Tris(hydroxymethyl)-aminomethan hydrochloride) pH 8; 1% Nonidet P-40; 4% protease inhibitor). Cell lysates were sonicated for 1 min, centrifuged at 11,900× *g* for 5 min at 4 °C. Aliquots were used to quantify the protein amount by Bradford protein assay [39]. All samples were stored at −80 °C until further analysis.

### 2.6. Cellular Efflux Experiments

For cellular efflux experiments, 2 × 10^6^ cells/well were seeded in 6-well plates and grown for 24 h. Cells were incubated with substances as described for the cellular uptake experiments (see 2.5). After 1 h incubation at 37 °C and 5% CO_2_, supernatants were removed, cells were washed with PBS and incubated with curcumin-free MEM Complete for further 8 h at 37 °C and 5% CO_2._ After 8 h, supernatants were stored at −80 °C. Equal to the cellular uptake experiments, cells were washed with PBS, scratched off the plate, washed again with PBS and lysed with 20 µL lysis buffer. Cell lysates were sonicated for 1 min, centrifuged at 11,900× *g* for 5 min at 4 °C and aliquots for protein quantification were stored with all other samples at −80 °C until further analysis. To observe curcumin efflux over time, representatively, efflux experiments were conducted with native and micellar curcumin as described above with a pre-incubation for 1 h and further incubations with curcumin-free MEM Complete for 6, 8, and 24 h.

### 2.7. Curcumin Quantification

For quantification of curcumin in cell lysates, supernatants, and stability controls, curcumin was extracted as described before [12]. Because no significant amounts of conjugated curcumin were detected in preliminary uptake and efflux experiments, no enzyme treatment was applied for deconjugation of phase II metabolites. To confirm these observations, curcumin was quantified with and without enzyme treatment in experiments on time-dependent efflux of native and micellar curcumin. For enzyme treatment, 1 mL sample was mixed with 10 µL 6 µmol/L HCl and 100 µL β-glucuronidase type H-1 from *Helix pomatia* (10 mg/mL in 0.1 M sodium acetate) for 45 min at 37 °C. For curcumin extraction from all samples on ice, 3 mL of extraction solvent (95% ethylacetate, 5% methanol) were added and mixed for 30 s. After a centrifugation at 1690× *g* for 5 min at 4 °C, 2 mL of the upper layer were transferred to a clean tube. The extraction was performed three times. After the second and third extraction, 3 mL of the upper layer were collected. Combined supernatants were evaporated and resuspended in 75 µL methanol for HPLC analysis. Next, 20 µL of each sample were injected into a Shimadzu-HPLC system (CBM-20A, SIL-20AC HT, LC-20 AT, DGU-20A 3R, Shimadzu Corporation, Kyoto, Japan) equipped with a Reprosil-Pur C18 AQ column (150 mm × 4 mm, 3 µm particle size; Dr. Maisch GmbH, Ammerbuch-Entringen, Germany) kept at 40 °C. Curcuminoids were quantified with a fluorescence detector (excitation/emission 426 nm/536 nm, RF-20A). Mobile phase (55% H_2_Odd adjusted to pH 3 with perchloric acid and 45% acetonitrile) was delivered at a flow rate of 1.4 mL/min. External standards for curcumin (purity ≥ 97.2%; CAS#458-37-7; Chromadex, Irvine, CA, USA), demethoxycurcumin (purity ≥ 98.3%; CAS#22608-11; Chromadex, Irvine, CA, USA) and bis-demethoxycurcumin (purity ≥ 99.4%, CAS#24949-16; Chromadex, Irvine, CA, USA) were used. All peaks were integrated with LabSolutions software (version 5.82, Shimadzu Corporation, Kyoto, Japan).

### 2.8. Statistical Analysis

All statistical analyses were conducted with GraphPad Prism 9 (version 9.0.0, GraphPad Software, San Diego, CA, USA). Data are presented as arithmetic mean ± SD or SEM, as indicated. All experiments were conducted in biological triplicates (*n* = 3), each consisting of technical duplicates. A one-way ANOVA and Dunnett’s post-hoc test with the medium control group serving as the reference, were used to compute differences between group means of P-gp activity. Differences between the formulations, regarding intra- and extracellular curcumin concentrations, in uptake and efflux experiments were assessed by one-way ANOVA, followed by Tukey’s post-hoc tests. The same tests were applied to assess differences between different time points, within the formulations, in time-dependent efflux experiments with native and micellar curcumin. Curcumin stability was confirmed by testing for differences between concentrations before and after 1 h incubation by *t*-tests. For all statistical tests, differences were considered significant at *p* < 0.05.

## 3. Results

### 3.1. Cell Viability

**Figure A1:**
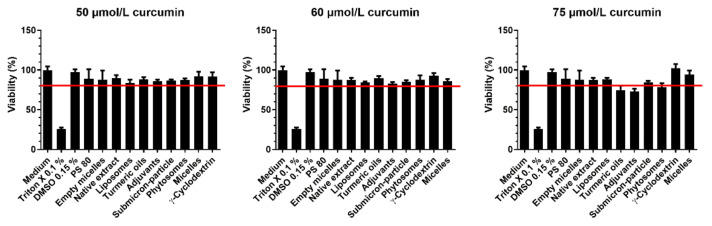
Mean (±SEM) viabilities (% of medium control) of LS180 after 1 h incubation at 37 °C and 5% CO_2_ with native curcumin or one of the seven formulations normalized to 50, 60, and 75 µmol/L curcumin. Medium control (100% viability) served as a reference, DMSO as the solvent control and Triton X 0.1% as the positive control. The red line shows 80% viability. PS 80, polysorbate 80.

Cell viabilities were not decreased below 80%, compared to medium control after incubating the cells for 1 h, with all galenic formulations normalized to concentrations of up to 60 µmol/L curcumin or the corresponding solvent controls, whereas at higher concentrations, some formulations decreased cell viability (Appendix A, Figure A1). Consequently, the formulations were normalized to a maximum of 60 µmol/L curcumin for further experiments.

### 3.2. P-Glycoprotein Activity

**Figure 1:**
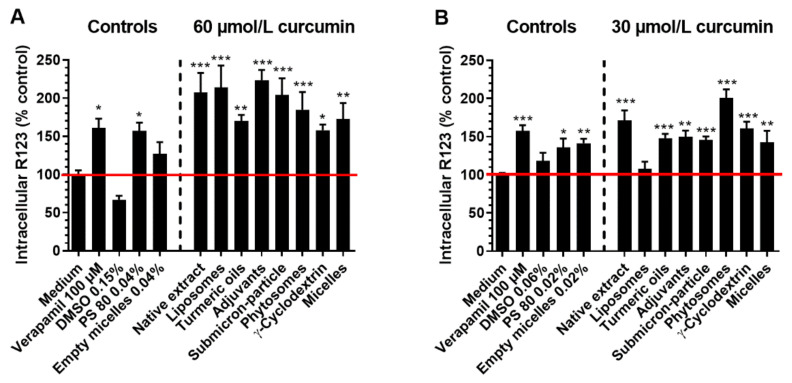
Intracellular accumulation (values > 100% indicate inhibition of P-gp activity) of the P-glycoprotein substrate rhodamine 123 (R123, in % to medium control), after co-incubation of LS180 cells with 1 µg/mL R123, and formulations normalized to: (**A**) 60 µmol/L or (**B**) 30 µmol/L curcumin at 37 °C and 5% CO_2_. Medium served as a negative control, verapamil as a positive control, DMSO, PS80, and empty micelles as solvent controls. All data are presented as mean ± SEM, compared to the medium control, as 100% (red line). All experiments were conducted in biological triplicates (*n* = 3) consisting of technical duplicates. * Difference at *p* < 0.05; ** difference at *p* < 0.01; *** difference at *p* < 0.001 (one-way ANOVA with Dunnett’s post-hoc test). PS80, polysorbate 80.

After the co-incubation of LS180 cells with the P-gp substrate R123 and 60 µmol/L curcumin from the different formulations, intracellular R123 accumulation was significantly increased compared to medium control (Figure 1A). Consequently, all formulations inhibited P-gp activity. In concentrations equal to the ones in curcumin micelles, pure PS80 (*p* = 0.0290) inhibited P-gp activity, but not when formulated as empty PS80 micelles (*p* = 0.7851). In concentrations of 30 µmol/L curcumin (Figure 1B), all formulations, except liposomal curcumin (*p* = 0.9956), significantly increased R123 accumulation.

In addition, PS80 (*p* = 0.0116) and empty PS80 micelles (*p* = 0.0022) increased intracellular R123. The solvent control DMSO had no effect on P-gp activity at any of the concentrations tested (Figure 1A,B).

### 3.3. Cellular Uptake and Stability of Curcumin

**Figure 2:**
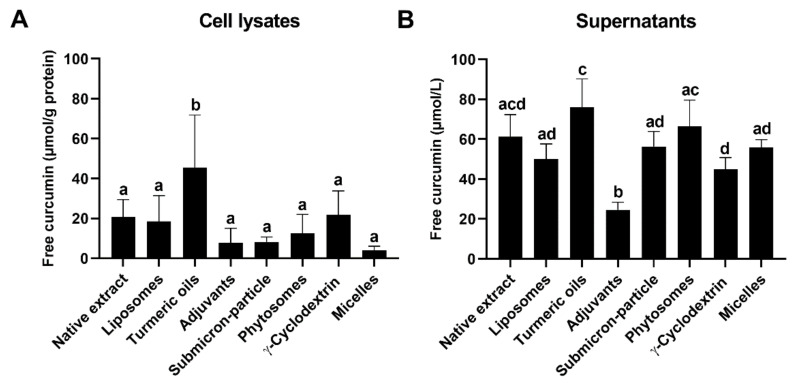
Free curcumin concentrations in (**A**) cell lysates in µmol/g protein and (**B**) supernatants in µmol/L after incubation of LS180 cells, with all formulations normalized to 60 µmol/L curcumin for 1 h at 37 °C and 5% CO_2_. All data are presented as mean ± SD. All experiments were conducted in biological triplicates (*n* = 3) and technical duplicates. Bars not sharing a common letter differ significantly at *p* < 0.05, consequently bars with at least one common letter do not differ significantly at p < 0.05 (one-way ANOVA with Tukey’s post-hoc test).

After 1 h incubation of LS180 cells, with formulations normalized to 60 µmol/L curcumin, intracellular curcumin concentrations were significantly higher when it was combined with turmeric oils compared to all other formulations (Figure 2A). No differences were observed between all other formulations.

**Figure 3:**
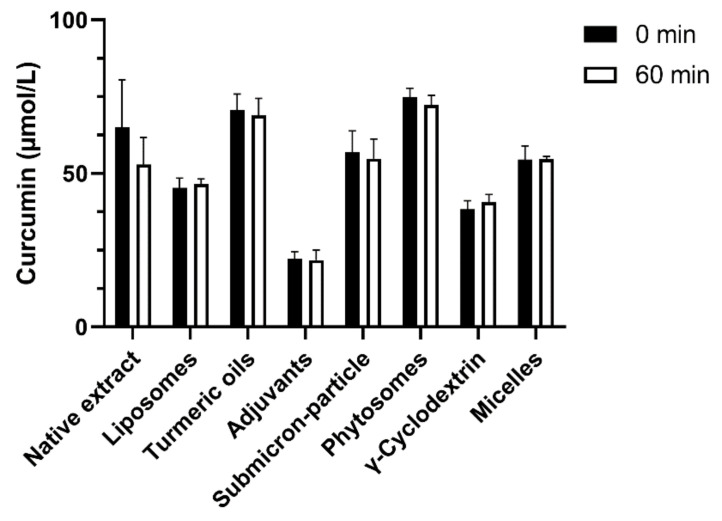
Curcumin concentrations (µmol/L) were quantified in MEM Complete, before and after 60 min, at 37 °C and 5% CO_2_ in the absence of cells to investigate curcumin stability. Data are presented as mean ± SD. All experiments were conducted in biological triplicates (*n* = 3) and technical duplicates. No significant differences at *p* < 0.05 were observed within each formulation between 0 min and 60 min (unpaired *t*-test).

Concerning the curcumin concentrations in the supernatants, significant differences were observed between individual formulations, but no formulation showed a clear effect (Figure 2B). Mainly, curcumin with turmeric oils reached significantly higher and, with adjuvants, significantly lower concentrations compared to the other formulations (Figure 2B). During the incubation for 1 h, curcumin, in form of all formulations diluted in cell culture medium, was stable, whereas baseline concentrations of the dilutions varied between 20 µmol/L and 65 µmol/L (Figure 3).

### 3.4. Curcumin Efflux

**Figure 4:**
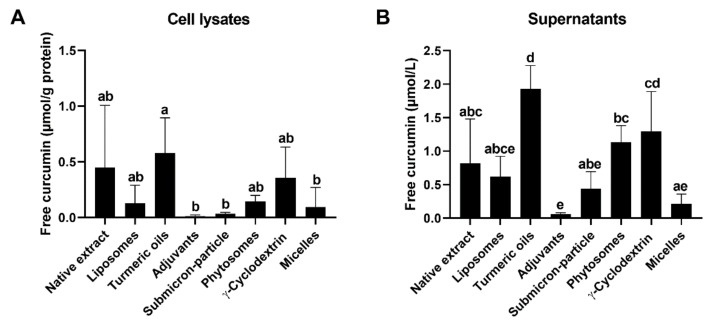
Free curcumin concentrations in (**A**) cell lysates (µmol/g protein) and (**B**) supernatants (µmol/L) after pre-incubation of LS180 cells for 1 h, with all formulations normalized to 60 µmol/L curcumin, and further incubation for 8 h, with curcumin-free cell culture medium at 37 °C and 5% CO_2_. Data are presented as mean ± SD. All experiments were conducted in biological triplicates (*n* = 3) consisting of technical duplicates. Bars not sharing a common letter differ significantly at *p* < 0.05, consequently bars with at least one common letter do not differ significantly at *p* < 0.05 (one-way ANOVA with Tukey’s post-hoc test).

After 8 h of incubation, intracellular curcumin concentrations were decreased from a range of 5 to 45 µmol/g protein, immediately after pre-incubation, with the formulations containing 60 µmol/L curcumin (Figure 2A) to concentrations lower than 0.5 µmol/g protein (Figure 4A).

**Figure 5:**
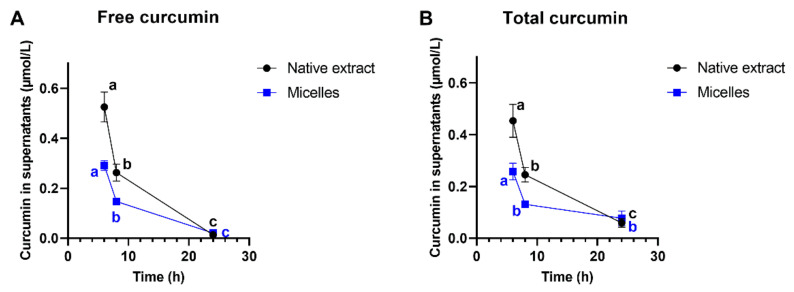
(**A**) Free and (**B**) total curcumin concentrations (µmol/L) in supernatants after pre-incubation of LS180 cells for 1 h, with native or micellar curcumin normalized to 60 µmol/L, and further incubation for 6, 8, and 24 h, with curcumin-free cell culture medium. Data are presented as mean ± SEM. All experiments were conducted in biological triplicates (*n* = 3) consisting of technical duplicates. Values within each formulation not sharing a common letter differ significantly at *p* < 0.05 (one-way ANOVA with Tukey’s post-hoc test).

Significant differences were observed between curcumin with turmeric oils and adjuvants (*p* = 0.0146), between curcumin with turmeric oils and submicron-particle curcumin (*p* = 0.0210), and between curcumin with turmeric oils and micellar curcumin (*p* = 0.0381; Figure 4A). The amount of curcumin in the supernatants after 8 h differed significantly between individual formulations (Figure 4B). Similar to observations after 1 h preincubation, mainly curcumin with turmeric oils showed increased (with adjuvants, decreased) concentrations (Figure 4B). Efflux experiments, over time, were conducted, representatively, for native and micellar curcumin. Free curcumin concentrations in supernatants decreased significantly from 6 to 8 h (*p* < 0.0001 for both formulations) and from 8 to 24 h (*p* < 0.0001 for micellar, *p* = 0.0002 for native curcumin; Figure 5A). After 24 h, concentrations were close to 0 µmol/L. We also quantified total (free + conjugated) curcumin concentrations (Figure 5B) and observed no differences to the values for free curcumin (Figure 5A). Consequently, no or negligible amounts of curcumin were conjugated.

## 4. Discussion

To the best of our knowledge, the present study is the first attempt to compare the effects of different galenic formulations of curcumin on the transport activity of P-gp. All curcumin formulations and native curcumin increased the accumulation of the P-gp-substrate R123 in LS180 cells when they were co-incubated for 1 h. P-gp inhibitory actions of unformulated curcumin were previously described for the intestinal cell lines LS180 and Caco-2 [23,24,26,27]. A dose-dependency, of the inhibitory effect of curcumin, on P-gp was observed for most of the formulations in the present study and is in agreement with earlier reports [24]. For example, liposomal curcumin significantly inhibited P-gp at a concentration of 60 µmol/L but not 30 µmol/L. Other formulations showed stronger inhibition at the higher concentration than at the lower concentration (Figure 1).

Consequently, the reported effects of native curcumin on the pharmacokinetics of drugs that are P-gp substrates [24,30,34], should also be considered and investigated in humans for formulated curcumin. Turmeric-derived turmerones, namely α-turmerone and aromatic turmerone, alone and in combination, were previously found to modulate P-gp activity, curcumin uptake, and transport through Caco-2 cells [18]. α-Turmerone inhibited, whereas aromatic turmerone induced P-gp activity. Co-incubation of curcumin with α-turmerone, but not aromatic turmerone, increased curcumin uptake and transport through Caco-2 cells. Both turmerones, in combination, led to intracellular curcumin accumulation but not to changes in curcumin transport [18]. Based on these observations, it was hypothesized that the administration of curcumin with turmeric oils, including turmerones, might increase its bioavailability. In the present study, curcumin with turmeric oils inhibited P-gp activity, but the extent of the effect was similar to all other formulations, including native curcumin (Figure 1). In agreement with this, we found no benefit of including turmeric oils in the formulation of curcumin in a recent crossover pharmacokinetic trial in healthy humans [13]. In contrast, the oral bioavailability of curcumin, in previous trials with healthy humans, was modestly higher (2-, 3-, or 8-fold) when turmeric oils were co-administered [11,40,41]. However, comparisons of fold-bioavailability of curcumin across published trials [14,19,42,43,44] should be made with caution, considering there is no linear relationship between the dose administered and blood concentrations achieved, as described in detail in Flory et al. (2021) [13]. Consequently, the present in-vitro data should be interpreted preferentially in the context of our previous human trial in which the same curcumin formulations were administered at identical doses of curcumin to all subjects [13].

We quantified the uptake of curcumin from the different formulations, into LS180 cells within 1 h, to better understand the similar P-gp-inhibitory activities of the formulations. Curcumin with turmeric oils showed significantly increased intracellular curcumin concentrations compared to all other formulations and native curcuma extract (Figure 2A), in agreement with previous observations [18]. All other formulations did not differ significantly. The comparable intracellular curcumin concentrations obtained for all formulations might explain the observed similarities in the inhibition of P-gp activity in the present in-vitro system, but they do not explain previously reported improvements in the bioavailability of curcumin in humans by its formulation. Especially, curcumin incorporated into polysorbate 80 micelles or a γ-cyclodextrin complex were up to 185-fold and 85-fold better bioavailable in-vivo, respectively [11,12,13,45], but it did not differ to other formulations or native curcuma extract, regarding uptake by intestinal cells in the present in-vitro study (Figure 2A), which is in agreement with previous observations in Caco-2 cells [13]. However, improvements in bioavailability for formulations compared to native curcumin, were reported to be 27-fold for submicron-particle curcumin [17], 20-fold for curcumin with piperine [20], and 9-, 13-, 18-, or 19-fold for phytosomal curcumin [11,14,40,42], formulations that did not show a higher relative bioavailability in our previous comparative human study using identical doses [13], and, as already described, did not differ in cellular uptake in LS180 (Figure 2A) and Caco-2 cells [13].

A theoretically possible explanation for differences in cellular uptake would be different rates of degradation of unformulated, or formulated, curcumin in cell culture medium. Curcumin is instable at neutral to alkaline pH and its degradation at pH 7.0 to 7.4, similar to the conditions in our experiments, was reported to occur within 1 h [46,47,48]. The addition of serum to cell culture medium, on the other hand, increased curcumin stability. Ninety percent of curcumin in serum-free cell culture medium were degraded already after 1 h. In contrast, <20% of curcumin were degraded after 1 h and 50% after 8 h when the cell culture medium was supplemented with 10% serum [47]. In our cell culture medium, 10% fetal calf serum was added and may have thus stabilized curcumin, as no degradation was observed within the first hour of incubation (Figure 3). Baseline curcumin concentrations (0 min), quantified immediately after the addition of the formulations to the cell culture medium, varied between 40 µmol/L and 65 µmol/L, as a result of differences in water solubility (Figure 3). Curcumin with turmeric oils and phytosomal curcumin, especially, showed higher baseline concentrations, so, consequently, more curcumin was available for cellular uptake. The curcumin concentrations in cell lysates (Figure 2A) and supernatants (Figure 2B), after 1 h incubation of the cells with the formulations, reflect the differences in baseline concentrations: curcumin with turmeric oils and phytosomal curcumin had the highest, and curcumin with adjuvants had the lowest concentrations in cells and supernatants. Consequently, curcumin concentrations in cell culture supernatants mainly result from curcumin solubility and stability in the culture medium, and only minor decreases are caused by cellular uptake of curcumin. Nevertheless, differences due to unavoidable, minor variations in doses cannot be fully excluded.

Efflux of curcumin and its conjugated metabolites from intestinal cells back to the intestinal lumen, facilitated by efflux transporters, has been proposed as a critical factor limiting its bioavailability [9,46]. The inhibition of the transporters P-gp or breast cancer resistance protein reduced the efflux of free or conjugated curcumin from Caco-2 cells [9]. Here, we compared efflux of free curcumin over 8 h in LS180 cells pre-incubated with curcumin formulations for 1 h. As expected, intracellular curcumin concentrations decreased several-fold over time, indicative of its efflux (Figure 2A and Figure 4A). Intracellular curcumin concentrations were higher when it was administered with turmeric oils, compared to some of the other formulations. Curcumin concentrations in the supernatants, which mirror the amount of curcumin secreted from the cells, differed between the formulations, but no consistent effect was observed (Figure 4B). Similar to the uptake experiments, curcumin administered with turmeric oils mainly showed higher and, with adjuvants, lower concentrations in cell lysates and supernatants (Figure 2 and Figure 4). Based on the present in-vitro data, curcumin efflux from intestinal cells does not concur with observations on bioavailability in humans. Based on the present in-vitro, and our previous in-vivo and in-vitro data [13], we conclude that stability during digestion and post-digestive solubility, but not cellular uptake and efflux, appear to be critical factors for the bioavailability of curcumin.

Curcumin concentrations in supernatants decreased significantly from 6 to 8 h and from 8 to 24 h (Figure 5). The opposite, namely an increase over time, would be expected due to a permanent efflux of curcumin from cells and accumulation in the cell culture supernatant. An interplay between curcumin efflux and its degradation is likely, based on the present data. In agreement with this, a reduction in curcumin in the cell culture medium from 10 µmol/L curcumin to around 5 µmol/L occurred within 8 h, even though the medium was supplemented with serum [47]. Our data suggests that degradation might become more important, and efflux less important, with time, thus explaining the decreasing curcumin concentrations (Figure 5).

Intracellular metabolism is another factor that might contribute to decreasing curcumin concentrations with time. As observed in human and rat intestinal and hepatic microsomes, curcumin is mainly conjugated to curcumin glucuronide and curcumin sulfate, but also reduced to dihydro-, tetrahydro-, hexahydro-, or octahydrocurcumin [49]. In human plasma, no free curcumin is detectable after oral administration, even at very high doses of up to 12 g curcumin [10]. Interestingly, in time-dependent efflux and preliminary uptake experiments, curcumin concentrations with (free + conjugated) and without (free curcumin) treatment with β-glucuronidase were equal in supernatants and cell lysates after 1 h incubation with native or micellar curcumin or, as shown in Figure 5, after 1 h pre-incubation with native and micellar curcumin and, further, 6, 8, and 24 h incubation with a curcumin-free medium. Consequently, and in contradiction to data on curcumin plasma concentrations from human trials [8,10,11,12,13,50], no or negligible amounts of glucuronidated or sulfated curcumin were detectable in cell culture medium, although the expression of the relevant enzymes (UDP-glucuronosyltransferase and sulfotransferase) in LS180 cells was reported previously [51,52]. In other publications reporting on curcumin uptake and transport through intestinal cells, no enzyme treatment was described and exclusively free curcumin was quantified [46,53]. Dempe et al., (2013) did not detect any curcumin glucuronide or sulfate in Caco-2 cell pellets after 3 h incubation with native curcumin. Curcumin glucuronide was observed in small amounts in the supernatants. After 1 h, no curcumin sulfate, small amounts of curcumin glucuronides, and, mainly, free curcumin were quantified in the basolateral and apical chambers [54]. Although UDP-glucuronosyltransferase and sulfotransferase isoforms, involved in curcumin metabolism, are expressed in Caco-2 cells [55], their activities in this cell line are low compared to other model systems [56]. Thus, the presently available cell culture systems do not appear to adequately reflect the metabolism of curcumin in humans.

## 5. Conclusions

In conclusion, native and formulated curcumin inhibited P-glycoprotein activity in cultured intestinal cells, without significant influences of the galenic formulation. Furthermore, cellular uptake by, and efflux from, intestinal cells were not significantly affected by its formulation, even though significant differences in the bioavailability of different curcumin formulations, front and foremost micellar curcumin, have been reported. Consequently, the presented data suggest that intestinal uptake and efflux may not be important regulatory points, limiting the bioavailability of the direct and indirect antioxidant curcumin. Nevertheless, the inhibition of P-glycoprotein activity by curcumin warrants further investigation, as it may pose the risk of undesired interactions with prescription drugs.

## Data Availability

The data presented in this study are available in the article. Raw data are available from the corresponding author upon reasonable request.
